# Epstein Barr virus associated venous thromboembolism

**DOI:** 10.55730/1300-0144.5998

**Published:** 2025-03-18

**Authors:** Yeliz ÇİÇEK, Ali MERT

**Affiliations:** 1 Department of Infectious Diseases and Clinical Microbiology, Faculty of Medicine, İstanbul Medipol University, İstanbul, Turkiye; 2 Department of Internal Medical Sciences, Faculty of Medicine, Medipol University, İstanbul, Turkiye

**Dear Editör**,

Epstein Barr virus (EBV) infection frequently manifests as either asymptomatic or flu-like symptoms. However, it may also result in mononucleosis syndrome, which is defined by hepatosplenomegaly, pharyngitis, cervical lymphadenopathy, hepatitis, atypical lymphocytosis, and monocytosis. EBV-associated venous thromboembolism (VTE) has rarely been reported in the medical literature [1–6]. According to our research, there are six case reports of EBV-associated thrombosis in the literature. Table summarizes information on six cases of EBV-associated thrombosis reported in the literature. In comparison, there are approximately 100 case reports of cytomegalovirus (CMV)-associated thrombosis in immunocompetent patients. Being from the same family might indicate that EBV has the potential for prothrombotic activity [7].

A 31-year-old male patient was admitted to our unit with complaints of fever, sore throat, weakness, fatigue, 12 kg weight loss in the last 20 days, and nausea and vomiting for the last three days. The center that referred the patient for evaluation detected lymphocytosis on the hemogram, splenomegaly (14 cm) on abdominal ultrasonography, and atypical lymphocytes in the peripheral blood smear. In the evaluation performed by the hematology department, the patient complained of pain and swelling in the right leg, and because no significant findings other than reactive lymphocytes were detected in the peripheral blood smear, the patient was consulted with us with a preliminary diagnosis of infection. The blood pressure was 110/70 mmHg, the heart rate was regular at 105 beats per minute, the respiratory rate was 20 breaths per minute, and the fever was 38.2 °C. On examination of the patient, the oropharynx was hyperemic and exudative, and crypts were occasionally observed. Multiple painful lymph nodes were palpable in the anterior cervical region. Compared with the left leg, the patient’s right leg was swollen, with no increase in temperature or redness, and the Homan’s sign was positive. Downey cells were seen in the patient’s peripheral blood smear as blue, large cytoplasm surrounded by erythrocytes. The heterophile antibody test was negative, and the EBV tests were compatible with acute EBV infection (The EBV VCA IgM was 24.92 U/mL (<0.5 U/mL is normal), and the EBV VCA IgG was 17.30 U/mL (<0.75 U/mL is normal)). The patient received serological testing for CMV, a virus recognized in the literature for its association with an elevated risk of venous thrombosis. CMV IgM was 0.08 (<0.7 negative), and CMV IgG was 35 (>6 positive) were identified. Two weeks later, the CMV IgG level was recorded at 36.2, and CMV syndrome was dismissed due to the lack of a 4-fold titer increase. History, clinical, and serological tests that do not match up, as well as other infectious agents that could cause mononucleosis (human immunodeficiency virus, toxoplasma, herpes simplex virus, mumps, mycoplasma) have been ruled out. The patient’s right lower extremity venous Doppler was reported as “thrombus filling the lumen was observed in the deep crural veins posterior to the cruris and is compatible with acute deep vein thrombosis.”

In this case, the patient did not have a history of malignancy, trauma, immobilization, chronic inflammatory disease, or central venous instrumentation that could have resulted in thrombosis. The most prevalent underlying autoimmune etiology of thrombosis was potentially ruled out by the negative test results for antinuclear, lupus, and anticardiolipin antibodies. Hereditary thrombophilia has not been taken into account, as neither the patient nor his family had a history of VTE, despite the inability to execute certain hereditary thrombophilia tests.

The patient’s complaints regressed, and he was removed from full-cure follow-up.

Primary EBV infection, which usually presents as an asymptomatic and self-limiting disease in children, may lead to an atypically severe clinical presentation in adults. VTE should be considered in the differential diagnosis of all adult patients presenting with signs and symptoms suggestive of primary EBV infection, and EBV should be recognized as a potential trigger for VTE.

## Figures and Tables

**Table:** Characteristics of patients complicated with thrombosis due to EBV infection.

Author	Age	Sex	Complication	Predisposing factors
Yamazaki et al. [1].	25	F	DVT and PE	Positive antiphospholipid antibody
Husein et al. [2].	21	M	Cerebral vein thrombosis	Positive antiphospholipid antibody
Zaher et al. [3].	29	M	hepatic veins thrombosis (Budd-Chiari syndrome)	Positive antiphospholipid antibody
Hyakutake et al. [4].	27	M	Portal vein thrombosis	No information
Bader et al. [5].	61	M	DVT and PE	No information
Maier-Stocker et al. [6].	22	M	Complete thrombosis of the right internal jugular vein (Lemierre’s syndrome)	No information

* F = Female, M = Male, DVT = Deep vein thrombosis, PE = Pulmonary embolism
